# Combined surgical excision with skin grafting and thermal therapy for refractory plantar warts: a therapeutic approach in immunocompromised patients

**DOI:** 10.1093/jscr/rjaf833

**Published:** 2025-10-17

**Authors:** Yong Tang, Li Zhang, Bai-Jie Lin

**Affiliations:** Department of Burn and Plastic Surgery, Chengdu Second People's Hospital, 10 Qingyun South Street, Chengdu, Sichuan 610017, China; Department of Dermatovenereology, Chengdu Second People's Hospital, 10 Qingyun South Street, Chengdu, Sichuan 610017, China; Department of Thoracic and Cardiovascular Surgery, Chengdu Second People's Hospital, 10 Qingyun South Street, Chengdu, Sichuan 610017, China

**Keywords:** recalcitrant warts, surgery, skin grafting, thermal therapy, immunosuppressed patients

## Abstract

Refractory cutaneous viral warts in the immunocompromised carry a high risk of recurrence and malignant change. Evidence for effective treatment is scarce. A 54-year-old woman with systemic lupus erythematosus, steroid-induced diabetes, and multiple comorbidities presented with plantar warts resistant to topical agents, cryotherapy and laser therapy for a decade. Progressive ulceration, pain, and impaired gait made surgery unavoidable. Histology confirmed benign verrucous change. Two-stage management comprised radical excision and negative-pressure wound therapy, followed by split-thickness grafting. On healing, daily 44°C foot soaks for 30 min were prescribed. At 6 months, warts had resolved, pain ceased, mobility returned and there was no recurrence. Radical excision, immediate grafting and adjunctive hyperthermia delivered durable remission in this high-risk patient. This regimen warrants prospective comparison with conventional treatments in immunocompromised patients with recalcitrant warts.

## Introduction

Cutaneous verrucae vulgaris are benign lesions brought on by epidermal human papillomavirus (HPV) infection and are often refractory to treatment in immunocompromised patients. In this group the lifetime risk of HPV-driven cutaneous malignancy and therapy-resistant warts rises [1]. Complications are both more common and more severe, so regimen choice must weigh delayed wound healing and adverse effects. Available treatments for periungual warts include salicylic acid, cryotherapy, laser therapy, topical imiquimod, intralesional bleomycin, and immunotherapy [2]. None, however, reliably ensures complete or sustained remission of recalcitrant lesions [3]. We describe an immunocompromised patient with recurrent plantar warts in whom sustained clearance was achieved and no recurrence has been seen at 6 months.

## Case report

A 54-year-old woman presented with plantar warts resistant to treatment on both hands and feet for 10 years. Topical 5-fluorouracil, salicylic acid, cryotherapy, laser ablation, and traditional Chinese remedies had all failed. During the previous 24 months, ulceration, pain and impaired gait had worsened and caused marked psychological distress. A 20-year history of systemic lupus erythematosus (SLE), treated with long-term prednisone, had led to steroid-induced diabetes, stage 3 diabetic nephropathy, neuropathy and osteoporosis. Current medication comprised mecobalamin, miglitol, epalrestat, linagliptin, and high-dose pregabalin for neuropathic pain. She also had stage 3 hypertension, hypertensive heart disease, left-sided chamber enlargement, and previous lacunar infarcts.

**Figure 1:**
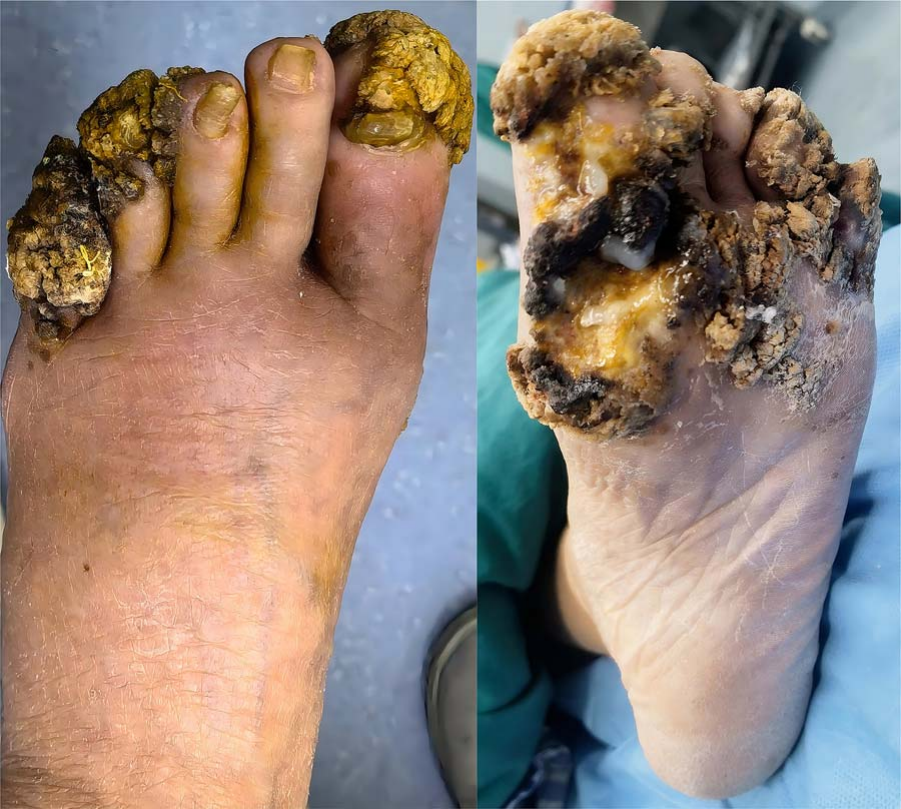
Extensive verrucae vulgares on the plantar and dorsal aspects of the left foot in a 54-year-old immunocompromised woman.

Hyperkeratotic nodules and ulcers involved the metacarpophalangeal and interphalangeal joints and both dorsal and plantar aspects of the left foot (Fig. 1). Cauliflower-like verrucous plaques with yellow-grey hyperkeratosis clustered across the soles and toes; the plantar surface also showed ulceration. Wound cultures yielded multidrug-resistant *Pseudomonas aeruginosa*; compromised perfusion, an unsuitable wound bed and the patient’s overall frailty necessitated a staged procedure.

Stage 1 entailed radical excision of plantar warts and ulcers to debulk the left foot. Each wart was shaved at the base and necrotic or granulation tissue excised until viable soft tissue was evident. Disease involved the fifth toe and exposed bone, prompting ray amputation after informed consent. Negative-pressure wound therapy (NPWT) was commenced immediately with dressing changes every 72 h. Post-operative analgesia comprised oral pregabalin and modified-release tramadol. Concurrently, we continued to monitor her comorbidities and provide supportive care. Histology confirmed a squamous papilloma with koilocytosis and hyperkeratosis consistent with viral wart, without dysplasia.

Stage 2 began once granulation was adequate: a split-thickness graft from the ipsilateral thigh was applied. NPWT continued to promote graft take. The left-foot lesion had been fully excised; the wound healed well, leaving only minor scar. Pain subsided completely and analgesics were stopped. Both stages were carried out under general anaesthesia with prophylactic antibiotics and close surveillance for infection. No complications arose post-operatively. All grafts had healed within 3 weeks. After healing, she was instructed to soak her foot in 44°C water for 30 min daily.

**Figure 2:**
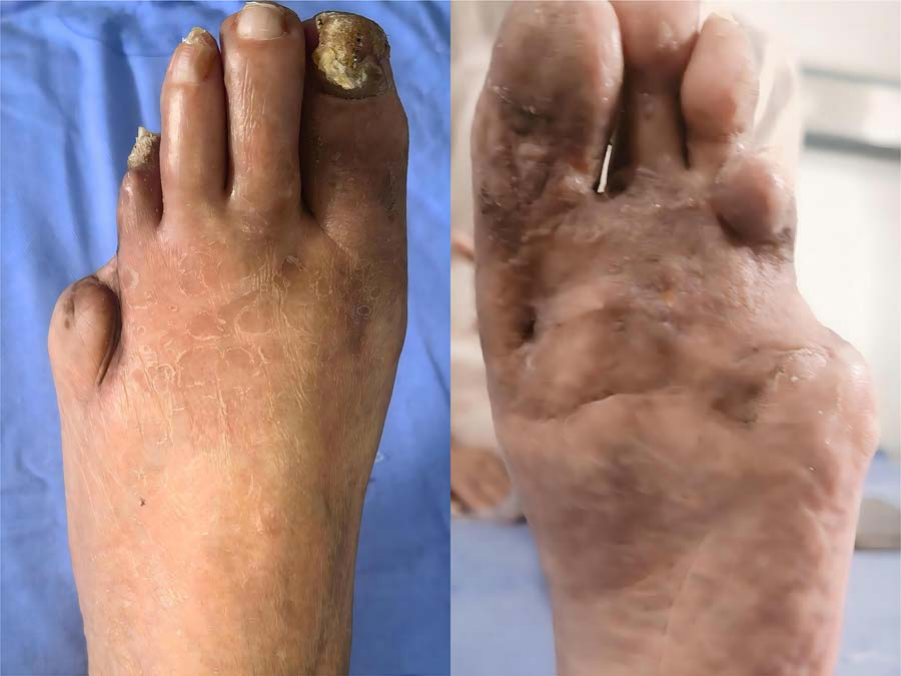
Left foot of the same patient 6 months after two-stage excision, split-thickness grafting and 44°C hyperthermia, showing complete remission, and minimal scarring.

No recurrence has been noted at 6 months (Fig. 2). She reported normal mobility and a clear improvement in quality of life.

## Discussion

The burden and severity of HPV warts rise with the level of immunosuppression, notably in SLE. These patients carry a greater lifetime risk of HPV-driven malignancy of the tongue, skin, and cervix [1]. Endogenous glucocorticoids, as seen in Cushing’s syndrome, lower lymphocyte counts, blunt activation, and hasten apoptosis [4]. High-dose steroids further deplete B and T cells and blunt pattern recognition receptor- and cytokine-receptor signalling, heightening infection susceptibility [5]. Together these factors explain the therapeutic challenge presented by this patient.

Available agents range from salicylic acid and cryotherapy to laser ablation, topical imiquimod, intralesional bleomycin and immunotherapy [2, 6]. No single modality delivers durable clearance [3], and adverse events such as pain, infection or nail dystrophy are common, especially in the immunocompromised. Combined approaches exist: superficial shaving with photodynamic therapy has shown benefit for recalcitrant periungual lesions [7], topical 3% cidofovir has cleared lesions in post-transplant severe combined immunodeficiency recipients [8], and surgical excision followed immediately by Nd:YAG laser has yielded high clearance rates in immunocompromised hosts [9], yet these cohorts were less immunosuppressed and had smaller lesions than our patient.

Her immune compromise, persistent ulceration and malignant potential warranted radical excision once conventional measures failed. Two-stage excision and grafting eradicated warts and ulcers, abolished pain and restored mobility whilst lowering the risk of autoinoculation.

Hyperthermia has shown efficacy against plantar warts in immunosuppressed hosts [10–12]. Heat absorption is temperature- and time-dependent; ≥ 43°C triggers apoptosis and may restore HPV-specific immunity [13, 14]. Earlier work recorded 44°C soaks as well tolerated and capable of mobilizing Langerhans cells to lymph nodes, thereby enhancing antigen presentation and anti-HPV T-cell activity [10]. We therefore advocate hyperthermia as a simple, effective adjunct after radical excision. Post-operatively, daily 44°C foot soaks were prescribed for prophylaxis.

Our goal was durable wart clearance to prevent autoinoculation, malignant transformation, pain and functional loss. Although relapse is common in immunocompromised patients, adjunctive hyperthermia is expected to lower the risk relative to excision alone and to yield milder recurrences that do not compromise daily life. In this complex case, radical excision with split-thickness grafting followed by immediate 44°C foot soaks achieved rapid, complete remission without severe complications. This combined strategy appears effective, expeditious, and safe for recalcitrant warts in immune-suppressed hosts.

Although this single case achieved sustained remission, its evidentiary weight is limited; multicentre prospective studies are required to validate the safety and efficacy of staged excision plus hyperthermia in immunocompromised patients with refractory warts.

## Data Availability

Data sharing is not applicable to this article, as no datasets were generated or analyzed in the current study.
